# Anti-Tumor Necrosis Factor-Alpha Associated Kaposi Sarcoma in a Patient with Ulcerative Colitis: A Case Report and Literature Review

**DOI:** 10.34172/mejdd.3418

**Published:** 2026-04-30

**Authors:** Reza Fatemi, Zeinab Mahabadi, Seyed Aram Fatemi, Sania Alizadeh, Binazir Khanabadi, Pardis Ketabi Moghadam

**Affiliations:** ^1^Research Institute for Gastroenterology and Liver Diseases, Shahid Beheshti University of Medical Sciences, Tehran, Iran

**Keywords:** Kaposi sarcoma, Tumor necrosis factor-alpha inhibitors, Infliximab, Ulcerative colitis, Adverse drug reaction, HHV-8

## Abstract

Kaposi sarcoma (KS) is a rare vascular malignancy associated with human herpesvirus-8 (HHV-8). Tumor necrosis factor-alpha inhibitors (anti-TNF-α agents) are widely used in inflammatory bowel diseases such as ulcerative colitis (UC). While these biologics are effective, they carry a risk of opportunistic infections and, rarely, malignancies. We report a rare case of KS developing in a patient with UC following infliximab treatment. A 67-year-old man with a 3-year history of moderate to severe UC, maintained on azathioprine and mesalazine, initiated induction therapy with infliximab (four 100 mg vials per dose). One week after the first infusion, he developed lower limb edema. Four months later, he presented with multiple violaceous cutaneous plaques. A skin biopsy confirmed KS, and HHV-8 serology was positive. Infliximab was immediately discontinued. The patient subsequently received systemic chemotherapy (liposomal doxorubicin) with complete resolution of the cutaneous lesions. This case highlights KS as a severe, albeit rare, adverse event of anti-TNF-α therapy, emphasizing the need for vigilance, prompt diagnosis, and intervention.

## Introduction

 Tumor necrosis factor-alpha inhibitors (anti-TNF-α agents) such as infliximab, adalimumab, and golimumab are cornerstone therapies for patients with moderate-to-severe ulcerative colitis (UC) who are refractory to conventional treatment.^1^ By modulating the immune response, they effectively reduce inflammation and induce remission. However, immunosuppression increases susceptibility to infections and may disrupt immune surveillance against virally-induced malignancies.^2^ Kaposi sarcoma (KS), caused by human herpesvirus-8 (HHV-8), is one such malignancy. While predominantly reported in transplant recipients and patients with HIV/AIDS, its incidence is rising in the context of iatrogenic immunosuppression, including biological therapy.^3^ We present a case of infliximab-induced KS in a seropositive patient with UC, followed by a discussion on pathogenesis and clinical management.

## Case Report

 A 67-year-old Iranian man was referred to the gastroenterology clinic with a diagnosis of extensive UC in the last 3 years. He was initially started on oral mesalazine (3 g/day) and azathioprine (2 mg/kg/day) but experienced recurrent flares. Due to steroid-dependent disease, the decision was made to start anti-TNF-α therapy. Pre-treatment screening for tuberculosis (Quantiferon-TB Gold) and hepatitis B/C was negative. Notably, HHV-8 serology was not performed as it is not part of the standard screening protocols.The patient received his first induction dose of infliximab (5 mg/kg, approximately 400 mg total from four 100 mg vials) intravenously. One week later, he presented with bilateral pitting edema of the lower limbs. Renal, hepatic, and cardiac function tests were within normal limits. The edema was managed conservatively with diuretics and partially resolved. It was considered a possible delayed infusion reaction or fluid retention, a known side effect. Four months after initiating infliximab (following four total doses), the patient developed multiple, non-pruritic, violaceous, maculopapular lesions on his lower legs and ankles (Figure 1). The lesions progressed rapidly in size and number.

**Figure 1:**
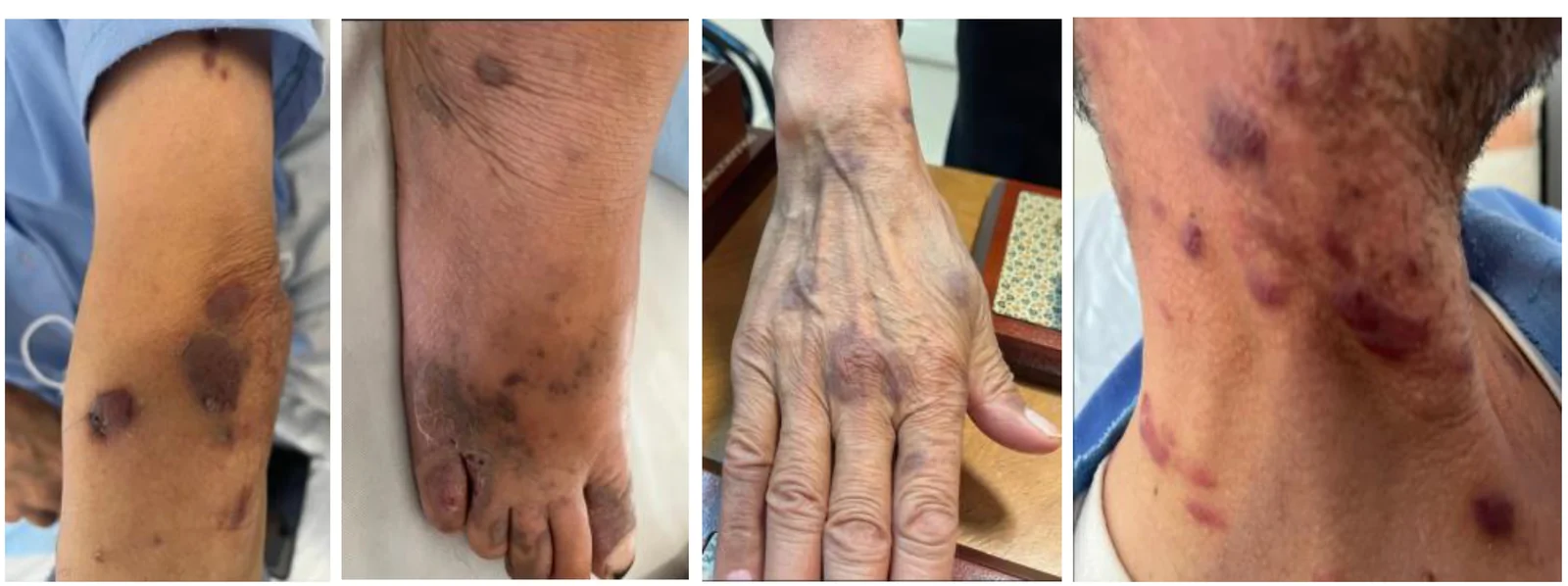
Clinical presentation showing multiple violaceous, infiltrated plaques on the lower extremities

 A punch biopsy of the most prominent lesion was performed. Histopathological examination revealed a dermal proliferation of spindle-shaped cells forming slit-like vascular spaces, with extravasated erythrocytes and hemosiderin deposition. Immunohistochemistry was positive for HHV-8 latency-associated nuclear antigen (HHV-8 LNA-1), confirming the diagnosis of KS (Figure 2). PCR from serum was positive for HHV-8 DNA.

**Figure 2:**
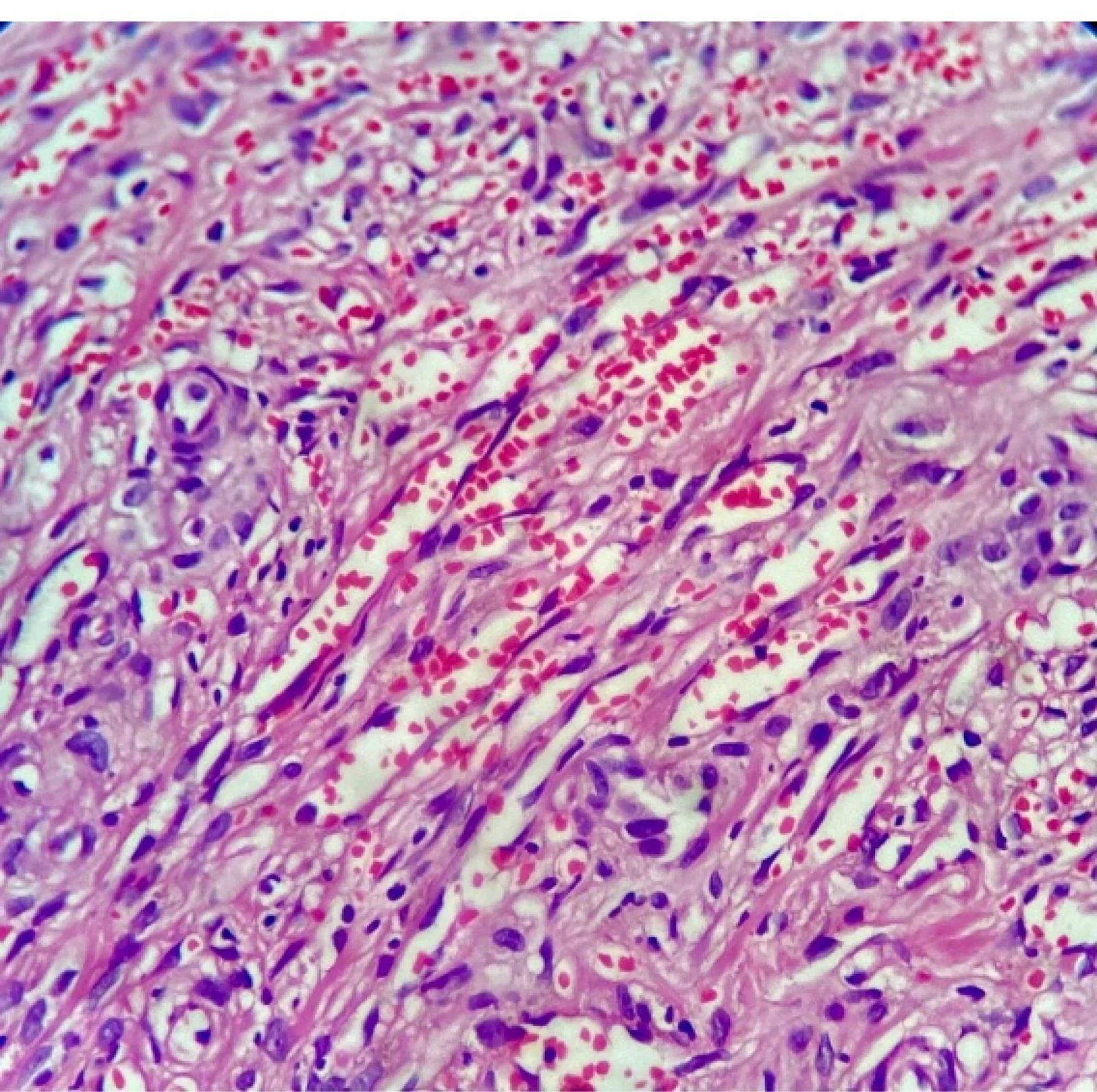
Histopathology of the skin biopsy. (A) H&E staining showing a dermal proliferation of spindle cells forming vascular slits (arrow) (200x). (B) Immunohistochemistry positive for HHV-8 LNA-1 in the nuclei of spindle cells (400x)

 Upon the diagnosis, infliximab was permanently discontinued. Azathioprine was also stopped. The patient’s UC activity was monitored closely. Staging workup for KS, including chest computed tomography (CT) and gastrointestinal endoscopy, revealed disease confined to the skin (classic cutaneous KS). The patient was referred to oncology and initiated on systemic chemotherapy with liposomal doxorubicin (20 mg/m^2^ every 3 weeks). After six cycles, a complete clinical response was achieved with the disappearance of all skin lesions (Figure 3). His UC remained in clinical remission on mesalazine monotherapy during a 12-month follow-up period, with no recurrence of KS.

**Figure 3:**
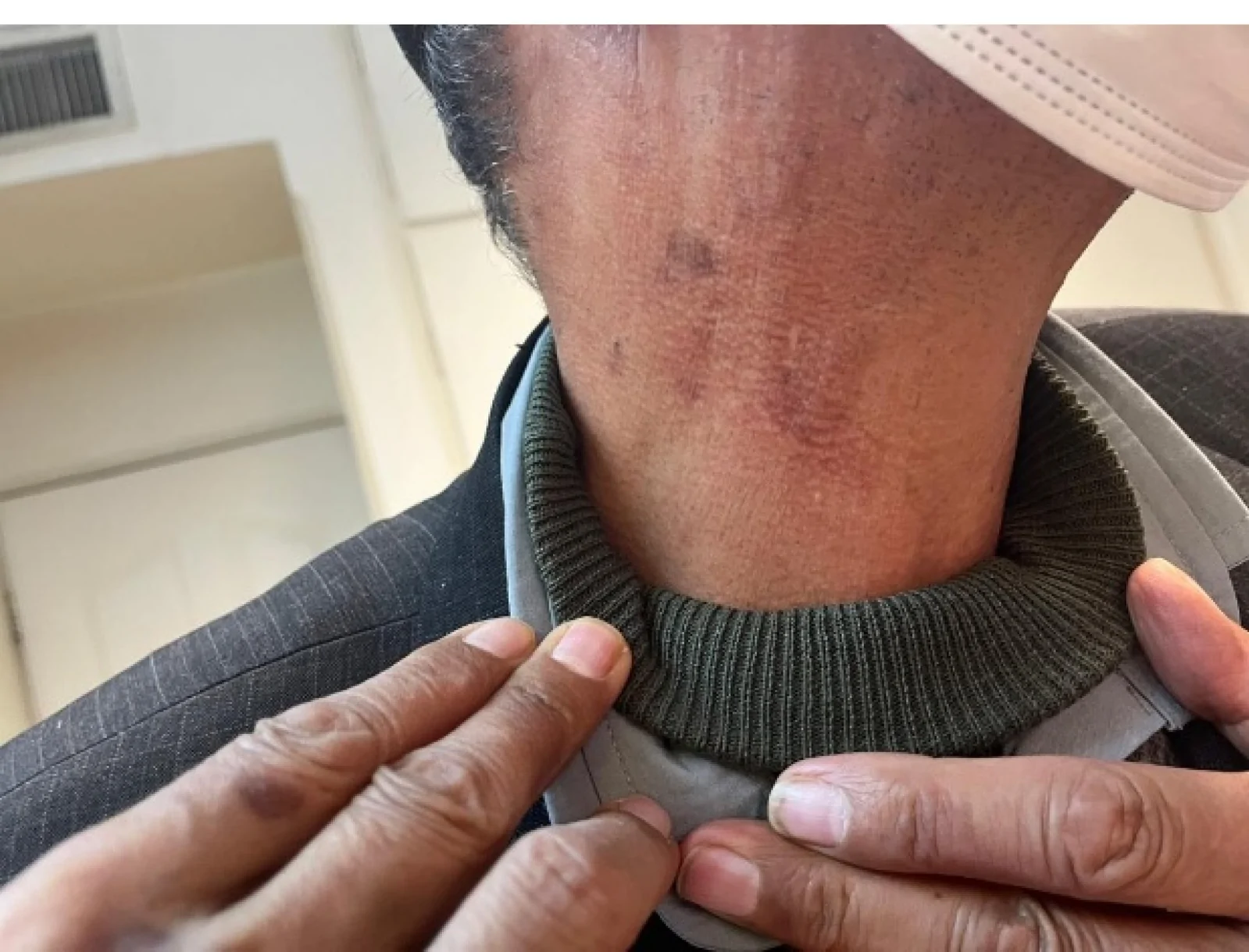
Disappearance of skin lesions after cessation of anti-TNF-α agent accompanied by six cycles of systemic chemotherapy with liposomal doxorubicin

## Discussion

 This case illustrates a severe paradoxical adverse effect of anti-TNF-α therapy: the development of HHV-8-associated KS. The temporal relationship between drug initiation and lesion appearance, coupled with the resolution after drug withdrawal and chemotherapy, strongly suggests a causative role for infliximab. The pathogenesis of anti-TNF-α-associated KS is multifactorial. TNF-α plays a complex role in oncogenesis. It can have both anti-tumor and pro-tumor effects. In the context of HHV-8 infection, TNF-α inhibition may impair the host’s immune surveillance against latent HHV-8, allowing viral reactivation and proliferation.^4^ Furthermore, anti-TNF-α agents may directly promote angiogenesis and the expression of vascular endothelial growth factor (VEGF), a key cytokine in the pathogenesis of KS.^5^ The concurrent use of azathioprine, a purine analog, likely contributed to the overall immunosuppressive burden.

 A review of the literature (PubMed, Scopus up to March 2024) using terms “anti-TNF,” “Kaposi sarcoma,” and “inflammatory bowel disease” reveals fewer than 30 reported cases worldwide, making this a rare complication. Most cases have been associated with infliximab and adalimumab in patients with rheumatoid arthritis or Crohn’s disease. Our case is among the few reported in a patient with UC.^6,7^ The initial presentation with lower limb edema is an intriguing and possibly under-recognized precursor. This may represent an early, non-specific inflammatory or angiogenic response preceding the overt vascular proliferation of KS.

 This case underscores several critical clinical implications:

1. **Pre-treatment Screening:** Routine screening for HHV-8 is not standard practice due to low seroprevalence in the general population and uncertain predictive value. However, in regions with higher HHV-8 endemicity or in high-risk patients, pre-treatment serology could be considered.
2. **Vigilance in Monitoring:** Clinicians must maintain a high index of suspicion for unusual cutaneous lesions in patients on biological therapies. Any new, violaceous, or persistent skin lesion warrants prompt dermatological evaluation and biopsy.
3. **Management:** The cornerstone of management is immediate discontinuation of the offending immunosuppressive agent. In many cases, this alone leads to regression of KS lesions.^7^ For persistent, extensive, or visceral disease, specific therapies such as liposomal doxorubicin (as used here), paclitaxel, or local radiotherapy are effective.
4. **Alternative Inflammatory Bowel Disease (IBD) Therapy:** After KS resolution, managing the underlying UC requires careful consideration. Non-immunosuppressive therapies or agents with different mechanisms of action (e.g., vedolizumab, ustekinumab, tofacitinib) should be prioritized.

## Conclusion

 Although Kaposi sarcoma is a rare complication of anti-TNF-α therapy, its potential severity necessitates awareness. This report adds to the limited literature on this association in UC. It highlights the importance of early recognition, definitive diagnosis via biopsy with HHV-8 testing, and immediate drug withdrawal. A multidisciplinary approach involving gastroenterologists, dermatologists, pathologists, and oncologists is essential for optimal patient outcomes. Further research is needed to identify risk factors and establish potential screening guidelines for vulnerable populations.
